# Objective Cervical Stiffness Assessment Using the Pregnolia System Prior to Induction of Labour: The CASPAR Feasibility Cohort Study

**DOI:** 10.1111/1471-0528.70229

**Published:** 2026-03-25

**Authors:** Elizabeth Medford, Steven Lane, Angharad Care, Andrew Sharp

**Affiliations:** ^1^ Liverpool Women's Hospital NHS Foundation Trust Liverpool UK; ^2^ University of Liverpool Liverpool UK

**Keywords:** cervical stiffness, induction, labour

## Abstract

**Objective:**

To determine if the Pregnolia System (PS) for cervical stiffness (CS) assessment is feasible for an induction of labour (IOL) cohort and to inform definitive study design for IOL outcome prediction.

**Design:**

Single‐centre, prospective, observational, feasibility study.

**Setting:**

Liverpool Women's Hospital (Liverpool, UK) IOL suite.

**Population:**

100 nulliparous, term pregnant women with intact membranes undergoing IOL.

**Methods:**

Participants underwent a CS assessment using the PS and routine Bishop Score (BS) before IOL commenced. Participants completed an acceptability questionnaire. Decision for IOL was as per clinical team and unit policy. Clinical outcomes were collected from electronic records after delivery.

**Main Outcome Measures:**

Feasibility outcomes; recruitment rate, acceptability profile and assessment tool fidelity in this cohort. Clinical outcomes; exploring associations between Pregnolia System CS results and IOL outcomes of interest.

**Results:**

Recruitment was good at 68% (100/148). Reliability analysis of completed CS assessments showed internal consistency to be excellent (Cronbach's Alpha 0.967). The PS assessment had a lower discomfort score compared to Bishop Score assessment (mean difference 3.73). Exploratory clinical outcome analysis revealed both cervical assessment tools had poor diagnostic capability for vaginal delivery following IOL (PS AUC 0.466 95% CI 0.340, 0.593, BS AUC 0.621 95% CI 0.497–0.745).

**Conclusion:**

This novel study confirms the feasibility of the PS for pre‐induction cervical assessment. However, it could not determine the clinical utility in this cohort due to the study design for feasibility outcomes.

## 1 Introduction

Induction of labour (IOL) is a common obstetric intervention affecting over one third of pregnant women in England [1]. Although subjective and only weakly predictive of delivery outcomes, the Bishop Score is still used in UK practice to determine whether cervical preparation is required [2, 3, 4, 5].

**FIGURE 1:**
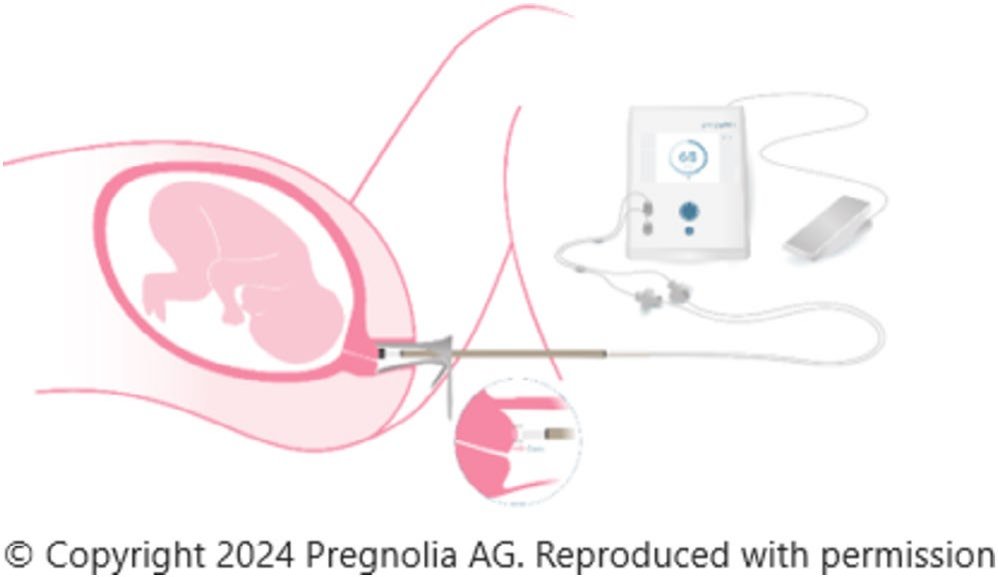
The Pregnolia System‐ vaginal probe and control unit with foot pump.

The Pregnolia System (PS) is a novel cervical assessment tool that provides cervical stiffness (CS) results through a vacuum‐aspiration technique [6, 7]. The assessment requires a speculum examination to visualise the cervix and allow placement of the sterile device probe directly to the anterior lip of the cervix (Figure 1). A foot‐pump is activated, and the control unit generates a negative pressure which displaces the underlying cervical epithelium into the device probe tip. Once adequate tissue displacement is achieved, the device automatically releases the pressure and detaches from the cervix whilst generating a final closing pressure, termed the Cervical Stiffness Index (CSI, mbar). One complete assessment requires consecutive, triplicate measurements as per manufacturer guidance for study protocols. The higher the stiffness of the tissue, the higher the CSI result [7, 8].

A small study involving an unselected, pregnant cohort demonstrated that the PS provided safe, objective, quantitative CS results [7, 8]. Using silicone cervical models, the PS has demonstrated improved reliability and reduced inter‐observer variability over digital palpation of the cervix, which could be extrapolated as a comparison to the Bishop Score [6]. However, there is very limited data available using the PS within clinical obstetric settings [9, 10]. Current research is mainly focused towards preterm birth (PTB) risk stratification following preliminary evidence showing the PS detects cervical softening before cervical length shortening [7, 11, 12]. Formal assessment of this novel device as a pre‐induction cervical assessment tool has not yet been extensively investigated.

The objective of this study was to formally explore the feasibility of using the PS for pre‐induction cervical assessment. This study aimed to: (1) determine recruitment rate, (2) verify acceptability within the IOL cohort, (3) establish fidelity of CS triplicate measurements and (4) inform application of triplicate measurements in clinical practice. Clinical outcomes regarding labour and delivery were collected to allow provisional exploration of associations between pre‐induction CS assessments and clinical outcomes of interest. This was to inform hypothesis development for future definitive studies if the PS assessment was proven to be feasible in this cohort. All candidate clinical measures were compared with the Bishop Score recorded concurrently, which served as the reference standard.

## 2 Methods

### 2.1 Study Design and Population

This feasibility study was a single site, prospective, cohort study of 100 nulliparous women with a term, singleton pregnancy, with intact membranes, undergoing IOL at the Liverpool Women's Hospital (LWH) in the UK. Need for IOL was determined by their clinical team. Exclusion criteria included: known cervical pathology preventing correct Pregnolia probe placement, active vaginal/cervical infection or bleeding, and known congenital uterine or fetal anomalies (Figure S1). Participants were recruited at time of attendance for IOL. After providing informed consent, participants underwent CS assessment using the PS, followed by their routine pre‐induction digital vaginal examination to assess the Bishop Score.

CS assessment using the PS was completed as per manufacturer guidance for study protocols; three consecutive measurements during a single speculum examination. The manufacturer required repeated measurements to validate reliability within a clinical setting. There is limited previous data using this device within a patient population, especially within a term, IOL cohort. However, a decline in CSI values during repeat consecutive measurements is expected due to cervical elasticity [7, 13]. To account for this, a prolonged time interval (> 30 min) between measurements would be required to allow the cervical tissue to return to its native state between measurements. Ultimately, this is not practical for patients or clinicians within a clinical setting. Therefore, consecutive measurements were taken with an expectation of a reducing trend that has been seen in other limited clinical cohorts outside of IOL [7]. Planned statistical analysis of triplicate measurement reliability allowed for validation within an IOL cohort and informed ongoing interpretation of triplicate results within future research or clinical settings.

Participants completed a structured questionnaire at the end of all study procedures (Figure S1). Questionnaire responses generated discomfort scores for the speculum examination, PS assessment and Bishop Score assessment from identical Likert scales, rating discomfort from 1 to 10. Defining a discomfort score for the speculum examination separate to the PS assessment distinguished the participant experience of application of the Pregnolia probe to the cervix and displacement of cervical tissue during the vacuum‐aspiration assessment from the speculum examination. This allowed for distinct evaluation specific to the participant experience of the Pregnolia device to truly determine acceptability of this novel assessment compared to well‐established cervical assessment tools within general obstetric practice (speculum and digital vaginal examination). In all cases IOL was performed as per unit policy in an observational study design. Feasibility outcomes were collected by the research team at the time of recruitment and study procedures. Clinical outcomes were collected from electronic hospital records following delivery and relied upon clinical team documentation to accurately reflect ongoing IOL, labour and delivery events (Figure S1).

The Liverpool Babies patient and public involvement (PPI) group was a fundamental contributor to study design and ensured the study addressed a research question that was important to the cohort and involved study procedures that were acceptable to women undergoing IOL. A 12 month‐follow up focus group allowed for results to be disseminated to participants and provided feedback to the study team to inform further research in this cohort.

### 2.2 Study Outcomes

Clear feasibility outcomes were outlined within the published study protocol [14]. These aim to establish the ability to recruit to a study within the IOL cohort using this novel cervical assessment tool, determine acceptability of the PS in this population and setting, and assess the PS ability to achieve reliable CS triplicate measurements in this cohort and infer appropriate clinical application of the results. Induction and delivery clinical outcomes have been collected to identify any preliminary associations for clinical utility of the PS in this obstetric clinical setting. Together, these outcomes can inform study design for a future definitive outcome prediction study.

### 2.3 Statistical Analysis

For feasibility outcomes, recruitment rate, acceptability data and obtaining triplicate results were represented as descriptive statistics, primarily with frequency of observations (percentages), but also generating mean (SD) and median (IQR) as appropriate. Further acceptability profiling involved comparison of Likert‐scale discomfort scores by generating mean differences and 95% confidence intervals (CI). Internal consistency of the device for triplicate measurements was analysed using Cronbach's alpha (CA) with a value ≥ 0.9 denoting excellent internal consistency.

For clinical outcomes baseline characteristics of the participants were compared using descriptive statistics; however, formal hypothesis testing has not been performed due to the feasibility design of this study and the lack of a formal sample size calculation. The sample size was designed for feasibility outcomes and not powered for testing statistical significance between differences in clinical outcomes. Preliminary exploration of associations between CS results and continuous clinical outcomes has been performed using scatter plots and generating correlation coefficients (*R* value). Exploratory diagnostic performance of the PS results for categorical clinical outcomes has been demonstrated through receiver operating characteristic (ROC) curves with area under the curve (AUC) and 95% CIs being calculated. Associations and diagnostic performance for Bishop Score and clinical outcomes have been shown for comparison. All data was analysed using SPSS 29.01.

## 3 Results

### 3.1 Feasibility Outcomes

#### 3.1.1 Recruitment Rate

148 women were approached for inclusion in the study with 100 agreeing to participate, achieving a recruitment rate of 68% (100/148). 97 participants completed the study visit (Figure S2). There were no dropouts following recruitment.

#### 3.1.2 Acceptability Profile

Of the 48 eligible patients who declined study participation, the majority declined due to the requirement for an additional speculum to facilitate the study procedure (*n* = 28, 58%). The remaining group either declined due to general anxiety surrounding the IOL process and found study involvement too overwhelming at that time (*n* = 10, 21%), or they had a general disinterest towards any additional research involvement (*n* = 10, 21%).

90 participants (90%) completed the post‐study procedure questionnaire. The Bishop Score had the highest mean discomfort score (4.93/10), whereas the PS had the lowest (1.20/10). The discomfort score for the PS was significantly lower than both the speculum examination (mean difference 3.15, 95% CI 2.58–3.73) and the Bishop Score (mean difference 3.73, 95% CI 3.09–4.37) (Table S1 and Figure S3). The PS, which utilises vacuum aspiration to displace the cervix by 4 mm, was more tolerable than current routine obstetric examinations (speculum and digital vaginal examinations). However, the PS requires the use of a speculum to facilitate the assessment and cannot be completed independently to a speculum examination. Therefore, the speculum discomfort score can be taken as the most noticeable part of the overall CS assessment. Despite this, the speculum discomfort score still improves upon the Bishop Score discomfort score (4.35/10 vs. 4.93/10). Due to the order of study visit procedures, and without a control comparison group, we are unable to determine if the speculum has directly influenced the Bishop Score experience in this study protocol.

Acceptability of the PS appeared good: 69% (*n* = 62) of the participants did not notice the device taking a measurement during the speculum examination, and 92% (*n* = 83) indicated they would agree to use the tool again (Figure S3). Of those women who had experienced a smear test before (*n* = 61), almost all (*n* = 59, 97%) of them felt having the PS assessment was better than or similar to having a smear. This is a very comparable gynaecological examination given the inherent requirement of a speculum for a smear assessment. Overall participants found the PS assessment, including the speculum, the most tolerable cervical assessment tool prior to their IOL (*n* = 47, 52%).

At the 12‐month follow‐up focus group (*n* = 10), participants confirmed the PS cervical assessment tool was acceptable to them. They reiterated the importance and need for more information regarding predicting outcomes of IOL to assist with their decision‐making, specifically achieving a vaginal delivery.

#### 3.1.3 Pregnolia System Fidelity

##### 3.1.3.1 Triplicate Measurement Obtainability

Two participants were not eligible for PS assessment at the time of the study procedure. At least one CS measurement was achieved in 88 participants of the remaining 98 eligible participants (90%). 76 participants (78%) who underwent PS cervical assessment achieved full triplicate measurements for analysis as per study protocol. 14% of the eligible participants did not achieve triplicate measurements due to increased mucus and secretions not allowing an adequate seal to be achieved with the Pregnolia probe for vacuum aspiration. A variety of other reasons accounting for the remainder are outlined in Table S2.

##### 3.1.3.2 Triplicate Measurement Reliability

When analysing overall sequential triplicate measurements by the median value, the first CS result (CSI 1: 51 mbar, IQR 45) was consistently the highest, with each subsequent measurement demonstrating reduced values (CSI 2: 41 mbar, IQR 45, CSI 3: 39 mbar, IQR 43). This confirmed the expected trend of consecutive measurements due to the viscoelastic nature of cervical tissue [7, 13].

Given the established difference in CSI between sequential measurements in a triplicate set, we compared different combinations of PS measurements in our reliability analysis to inform clinical application of the decreasing results. Overall, the PS has excellent internal consistency for CS measurements, regardless of which combination of the triplicate results was used (CA > 0.9 throughout) (Table 1). These results support the use of the first CS result obtained as the final CS value for clinical application.

**TABLE 1:** Reliability analysis of the Pregnolia System for triplicate cervical stiffness measurements using Cronbach's Alpha.

CSI 1	CSI 2	CSI 3	Min	Max	Mean*	Median*	1st CSI achieved	Cronbach's alpha	*N* used (total = 97)
X	X	X						0.967	76
X	X							0.951	78
X		X						0.933	77
	X	X						0.968	78
			X	X				0.966	87
					X	X		0.997	81
X					X			0.978	79
	X				X			0.989	80
		X			X			0.984	79
X						X		0.966	79
	X					X		0.992	80
		X				X		0.985	79
X			X					0.969	84
X				X				0.981	84
	X		X					0.970	81
	X			X				0.984	81
		X	X					0.980	79
		X		X				0.970	79
			X		X			0.991	81
			X			X		0.985	81
				X	X			0.992	81
				X		X		0.987	81
X							X	1.00	84
	X						X	0.952	81
		X					X	0.930	79
			X				X	0.966	87
				X			X	0.981	87
					X		X	0.977	81
						X	X	0.965	81
X	X	X					X	0.978	76

*Note:* CSI1‐ 1st attempted measurement of sequential triplicate measurements, CSI2‐ 2nd attempted measurement of sequential triplicate measurements, CSI3‐ 3rd attempted measurement of sequential triplicate measurements, Min‐ minimum result obtained, Max‐ maximum result obtained, 1st CSI achieved‐ first measurement to be successfully obtained during full assessment, *Mean and median included if ≥ 2 measurements available from CSI1, 2 & 3.

### 3.2 Exploring Clinical Utility

Baseline characteristics for the cohort are detailed in Table 2. Participant demographics were similar when comparing by mode of delivery. A greater proportion of IOL for reduced fetal movements (RFM) (22.5% vs. 9%) and maternal medical disease (10% vs. 5%) resulted in caesarean section, whereas all IOL for fetal concern resulted in vaginal delivery (10% vs. 0%). Participants that required additional cervical ripening methods (i.e., Propess & Prostin) resulted in an increased proportion delivered by caesarean section (22.5% vs. 5%). Women who achieved a vaginal delivery following IOL had a shorter interval from rupture of membranes until delivery (709 ± 423 vs. 917 ± 440), shorter duration of oxytocin usage (minutes) (452 ± 387 vs. 703 ± 402) and shorter duration of 1st and 2nd stages of labour (minutes) (265 ± 226 vs. 359 ± 218, 100 ± 75 vs. 171 ± 96). (Definitions outlined in Table S3).

**TABLE 2:** Demographic, induction and labour characteristics for the CASPAR study population.

Baseline characteristics	Total cohort *n* = 97	Mode of delivery	
		Caesarean section *n* = 40	Vaginal delivery *n* = 57
Age (years)	29 ± 5.5	29 ± 6.5	29 + 4.7
Booking BMI	27 ± 5.9	28 ± 6.9	26 ± 5.0
Ethnicity			
White	76 (78%)	30 (75%)	46 (81%)
Black	6 (6%)	3 (7.5%)	3 (5.3%)
Asian	11 (11%)	5 (12.5%)	6 (10.5%)
Mixed	3 (3%)	1 (2.5%)	2 (3.5%)
Other	1 (1%)	1 (2.5%)	0
IMD decile			
1	44 (45%)	20 (50%)	24 (42%)
2	13 (13%)	7 (17.5%)	6 (11%)
Indication for induction			
Post dates	38 (39%)	13 (32.5%)	25 (44%)
Reduced fetal movements	14 (14%)	9 (22.5%)	5 (9%)
Large for gestational age	13 (13%)	5 (12.5%)	8 (14%)
Maternal request	3 (3%)	2 (5%)	1 (2%)
Advanced maternal age	2 (2%)	2 (5%)	0
Maternal medical disease	7 (7%)	4 (10%)	3 (5%)
Gestational diabetes	9 (9%)	4 (10%)	5 (9%)
Pre‐eclampsia/Pregnancy induced hypertension	4 (4%)	1 (2.5%)	3 (5%)
Fetal concern	6 (6%)	0	6 (10%)
Other	1 (1%)	0	1 (2%)
Gestation at induction (weeks + days)	40 + 0 (2 + 1)	39 + 5 (2 + 0)	40 + 1 (2 + 2)
37 + 0–37 + 6	7 (7%)	4 (10%)	3 (5%)
38 + 0–38 + 6	19 (20%)	5 (12.5%)	14 (24%)
39 + 0–39 + 6	22 (23%)	13 (32.5%)	9 (16%)
40 + 0–40 + 6	16 (16%)	7 (17.5%)	9 (16%)
≥ 41 + 0	33 (34%)	11 (27.5%)	22 (39%)
Cervical ripening method			
Propess only	77 (79%)	28 (70%)	49 (86%)
Propess + Prostin	12 (12%)	9 (22.5%)	3 (5%)
Balloon	0	0	0
Nil required	8 (8%)	3 (7.5%)	5 (9%)
Rupture of membranes			
Spontaneous	22 (23%)	8 (20%)	14 (25%)
Artificial rupture of membranes (ARM)	68 (70%)	26 (65%)	42 (74%)
Unable to ARM	3 (3%)	3 (7.5%)	0
ARM not indicated	3 (3%)	3 (7.5%)	0
Missing	1 (1%)	0	1 (1%)
Interval from rupture of membranes to delivery (mins)	788 ± 438	917 ± 440	709 ± 423
Oxytocin required			
Yes	72 (74%)	30 (75%)	42 (74%)
No	19 (20%)	4 (10%)	15 (26%)
Not indicated	6 (6%)	6 (15%)	0
Duration of oxytocin^a^ (mins) *N* = 90	543 ± 408	703 ± 402	452 ± 387
Induction onset to delivery (hours)	41 ± 18	39 ± 14	41 ± 22
Established in active labour			
Yes	78 (80%)	21 (53%)	57 (100%)
No	19 (20%)	19 (47%)	0
Length of active 1st stage^a^ (mins) *N* = 48	274 ± 225	359 ± 218	265 ± 226
Achieved fully dilated			
Yes	61 (63%)	4 (10%)	57 (100%)
No	36 (37%)	36 (90%)	0
Length of 2nd stage^a^ (mins) *N* = 56	105 ± 78	171 ± 96	100 ± 75

*Note:* Continuous variables represented as Mean ± SD if normally distributed, and median (IQR) if not‐normally distributed. Categorical variables represented as number (%).

^a^ Missing information from electronic maternity clinical records, number included as *N*.

**FIGURE 2:**
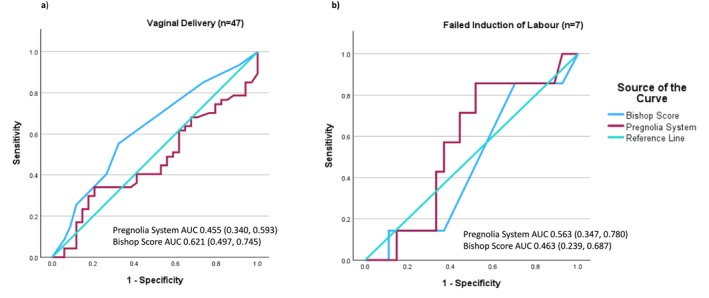
ROC curves to demonstrate predictive ability of Pregnolia System and Bishop Score for IOL outcomes; (a) Vaginal Delivery (b) Failed IOL. Failed induction of labour figures includes small numbers that this feasibility study is not powered for. Included for exploratory analysis purpose and potential hypothesis generation for future definitive studies.

The ability of PS (AUC 0.466, 95% CI 0.340–0.593) and Bishop Score (AUC 0.621, 95% CI 0.497–0.745) to predict vaginal delivery following IOL was poor, as an AUC ≤ 0.5 indicates no better performance than chance (Figure 2). Identifying those women who subsequently require a caesarean delivery after a failed IOL was determined to be a key clinical outcome of interest following IOL. Both PS & Bishop Score were poor predictive cervical assessment tools for this outcome (PS AUC 0.563, 95% CI 0.354–0.780 & Bishop Score 0.463, 95% CI 0.239–0.687). Similarly, there was no signal of diagnostic prowess for CS or Bishop Score for other IOL outcomes of clinical interest including achieving active labour, achieving fully dilated, spontaneous rupture of membranes, not requiring oxytocin and requiring more than one cervical ripening method (Figure S4 and Table S5). There was no clear association found between CS and duration of ruptured membranes (*R* = 0.042), duration of oxytocin (*R* = −0.104), interval from onset of induction to delivery (*R* = 0.081) or length of active 1st stage of labour (*R* = −0.215). The cervical assessment tool results also did not correlate with each other (*R* = −0.002). (Figure S5 and Figure S6).

This feasibility study protocol was able to collect outcome data to fulfil the core outcome set for trials on IOL (Table S4). However, collecting clinical outcome data in an observational study design relies upon clinical team documentation to accurately reflect pertinent study outcome data. As highlighted in Table 2, this study identified three key labour data points that contributed to missing clinical outcome data due to incomplete electronic records: (1) time commenced active labour, (2) duration of oxytocin and (3) time achieved full dilatation.

## 4 Discussion

### 4.1 Main Findings and Interpretation

The CASPAR study demonstrates that the use of the PS as a CS assessment tool at the time of IOL is feasible. However, its utility for IOL clinical outcome associations could not be demonstrated adequately due to the feasibility study design.

An adequate recruitment rate of 68% was achieved, which is comparable to other IOL studies where recruitment varies from 27% to 89% [9, 15, 16, 17]. Considering our study explores introduction of a novel assessment tool for this population, this recruitment rate reflects acceptability in this setting.

This study fully assesses participant experience and perception of the PS for cervical assessment as part of IOL. Participants preferred the CS assessment tool over the current reference standard of pre‐induction cervical assessment using the Bishop Score. From participant questionnaire results we recommend describing the assessment as ‘similar to or better than having a smear test’. Our comparison discomfort scores for the stiffness assessment and Bishop Score are supported by similar findings in a smaller preliminary study from Belgium [9]. Our study also captures perceptions regarding speculum examination at term gestation. Overall, 32% of eligible women declined participation, with 58% of these citing the requirement for a speculum examination as the primary reason. Although speculum examination is routine in gynaecological and obstetric practice, it is not commonly performed in the IOL setting, which may have influenced acceptability in this cohort. Among participants who underwent the procedure, the speculum examination had the highest reported discomfort score (4.35/10).

Although the PS itself was well tolerated, the requirement for a speculum examination represents an inherent limitation of the device and may pose a barrier to wider implementation. If the PS was demonstrated to provide clinically meaningful predictions of IOL outcomes, acceptability may improve with familiarity and clinical utility. However, the need for speculum examination remains an important consideration for scalability and real‐world implementation in obstetric practice.

This study has fully assessed obtainability of triplicate measurements at term gestation. There have been limited previous clinical studies using the PS at this point in pregnancy [7, 9]. Our study has highlighted several challenges in achieving triplicate measurements in this cohort, mostly due to difficulty with achieving an appropriate seal between the Pregnolia probe and the cervix secondary to increased vaginal secretions, mucus or discharge at this point in pregnancy. Salaets et al. [9] found an 11% prevalence of difficulty with vaginal mucus during use of the PS but did not fully explain how this impacted upon triplicate results and the final cohort used for analysis. Badir et al. [7] included the 1st measurement only in their reported analysis and didn’t evaluate obtainability of triplicate measurements. In the CASPAR study we have found a 14% prevalence of issues achieving an adequate seal for triplicate measurements; however, only 5% prevented no measurements at all. We obtained at least one CS reading in 90% of our cohort, but triplicate measurements in only 78%. The protocol requirement for triplicate measurements excludes 12% of the cohort with at least one available CS measurement. If this rule was applied clinically, 12% of the patients having this assessment performed prior to IOL would obtain results that could not be used. In comparison, 100% of participants or patients can achieve a documented Bishop Score.

By combining our obtainability and reliability findings, we have challenged the requirement for triplicate measurements when using the PS in either a study protocol or clinical setting. Our reliability analysis shows that despite observing differences in sequential measurements on each participant, the results remained strongly correlated and consistent across different combinations (e.g., first, second and third; highest vs. lowest; first measurement achieved vs. mean of all available). Given the observed difficulty obtaining triplicate results in the entire cohort, but ability to achieve at least one CS measurement, the reliability analysis supports the recommendation to use first CS measurement achieved during the assessment with the PS as the final CS result for that participant. This would improve the proportion of women with a meaningful result from 78% to 90%. Needing only one CS measurement will also reduce the duration required for CS assessment and consequently reduce speculum placement duration. This could potentially improve other factors that affect acquisition of results during this study procedure, such as tolerability of speculum and participant experience.

### 4.2 Strengths and Limitations

Study design was pragmatic to its clinical setting to directly assess potential for implementation within clinical practice. Data collection methods were robust and able to meet the requirements of the core outcome set for trials on IOL, again reflecting the ability of the study protocol to be upscaled to a larger definitive trial design. PPI group involvement took place throughout the study from protocol writing to dissemination of results and ensured the focus of study outcomes was centred to the patient's needs.

This study is limited by its size. Although this cohort size was adequate for ascertaining feasibility, which was the primary objective, it was not powered for clinical outcome associations and therefore cannot confirm clinical utility of the PS in the IOL population. However, our confirmation of feasibility and acceptability can inform appropriately powered larger studies that could answer the question of predictive acumen for IOL clinical outcomes.

This study did not provide an economic analysis of introducing the PS into routine pre‐induction clinical assessment. We would advise further studies evaluating this device include such an analysis.

### 4.3 Conclusion

This is the first study to explore the feasibility of using the PS for CS assessments in the IOL clinical setting. Our findings support the ability to adequately recruit to a clinical study using this new device within an NHS setting, confirm acceptability of this cervical assessment tool at the time of IOL in this population and provide clinical data to recommend the use of single CS measurements taken using the PS to improve measurement obtainability in this cohort. These findings confirm feasibility of the PS for cervical assessment prior to IOL in term, nulliparous women. At this time, no associations between pre‐induction CS assessment with the PS and outcomes of IOL have been demonstrated. Importantly, we also found no association between pre‐induction Bishop Score and IOL outcomes.

### 4.4 Practical Recommendations (Clinical and Research)

This feasibility study has confirmed that first‐time mothers facing the prospect of IOL want to know if the IOL process will achieve a vaginal delivery. Women were prepared to undergo a novel cervical assessment tool at the time of their IOL to try and answer this question. This confirms that predicting the mode of delivery following IOL is still a valid and relevant concern for our term obstetric population as well as having value for the organisation of hospital services. Further research within this field needs to keep this outcome at the centre of the question the research is going to answer.

We feel the feasibility of this device in this setting has been proven by the CASPAR study. If any further studies using the PS were conducted, they would need to be powered to give a definitive answer for the clinical outcomes associated with IOL. We would also recommend using the first CS measurements obtained using the PS as the final result for each participant.

## Author Contributions

**Elizabeth Medford:** conceptualization, methodology, validation, formal analysis, data curation, writing – original draft, visualisation, project administration. **Steven Lane:** methodology, formal analysis, writing – review and editing. **Angharad Care:** conceptualisation, methodology, writing – review and editing, funding acquisition, project administration, supervision. **Andrew Sharp:** conceptualisation, methodology, writing – reviewing and editing, project administration, supervision.

## Funding

Pregnolia AG provided the device for use in this study and provided financial aid to fund a Clinical research Fellow to undertake this research. Pregnolia AG had no role in the study design, decision to publish or preparation of the manuscript.

## Ethics Statement

Seasonal Research Ethics Committee, UK (23/LO/0627), 2/8/2023.

## Consent

All study participants have given written informed consent prior to entry to the study and were aware that participation is completely voluntary.

## Conflicts of Interest

The authors declare no conflicts of interest.

## Supporting information


**Figure S1:** CASPAR Study Flow Chart.

**Figure S2:** CASPAR Study Recruitment.

**Figure S3:** Comparison of Discomfort Score Between Study Procedures.

**Figure S4:** Comparison ROC curves between Cervical Assessment Tools for Induction of Labour Binary Clinical Outcomes.

**Figure S5:** Associations between Cervical Assessment Tools and Continuous Outcome of Interest.

**Figure S6:** Association between Cervical Assessment Tool Results; Bishop Score and Pregnolia System Cervical Stiffness.

**Table S1:** Participant Questionnaire Study Assessment Discomfort Scores.

**Table S2:** Summary of Reasons for not Achieving Triplicate Measurements.

**Table S3:** Definitions used for Clinical Outcomes.

**Table S4:** Feasibility of Collecting Core Outcome Set for Trials on Induction of Labour using CASPAR Protocol.

**Table S5:** Diagnostic Performance for Cervical Assessment Tools for each Binary Outcome of Interest.

## Data Availability

The data that support the findings of this study are available on request from the corresponding author. The data are not publicly available due to privacy or ethical restrictions.
