# Supplementary figures and images for: Objective Cervical Stiffness Assessment Using the Pregnolia System Prior to Induction of Labour: The CASPAR Feasibility Cohort Study
Source: BJOG. 2026 Mar 25;133(9):1762–70. doi: 10.1111/1471-0528.70229 (PMC13419266; doi:10.1111/1471-0528.70229)

**Figure S1**

*CASPAR Study Flow Chart*

**
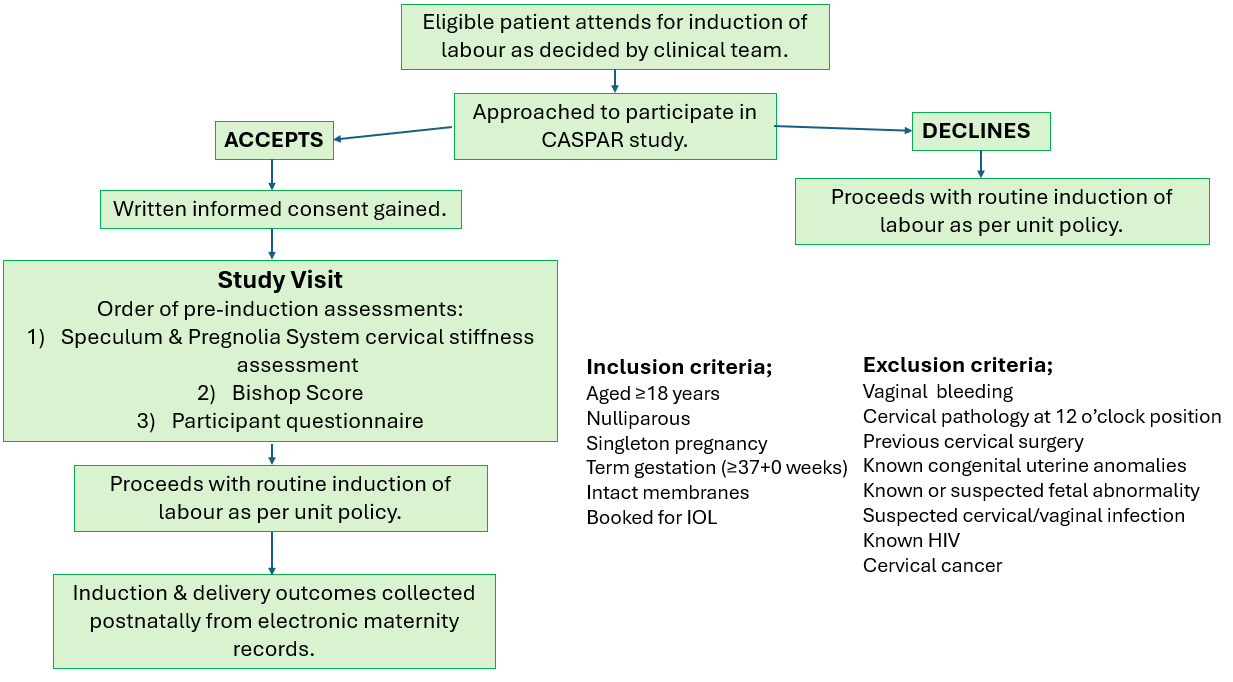
**

Supplement: Supplementary file 1 — Figure S1: CASPAR Study Flow Chart. [file BJO-133-1762-s002.docx]

**Figure S2**

*CASPAR Study Recruitment*


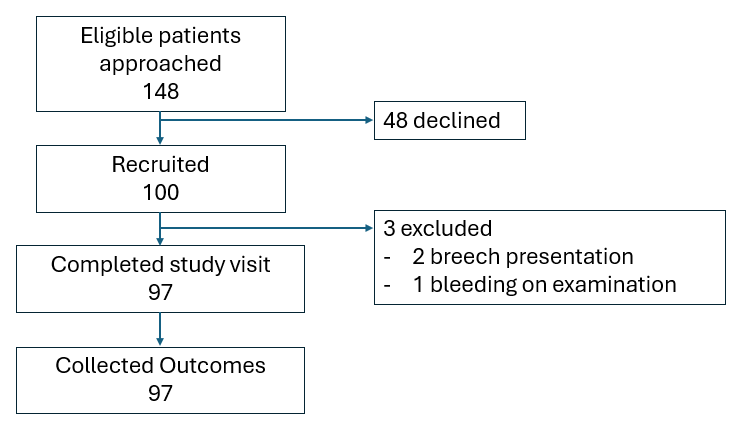

Supplement: Supplementary file 2 — Figure S2: CASPAR Study Recruitment. [file BJO-133-1762-s007.docx]

**Figure S3**

*Comparison of Discomfort Score Between Study Procedures*


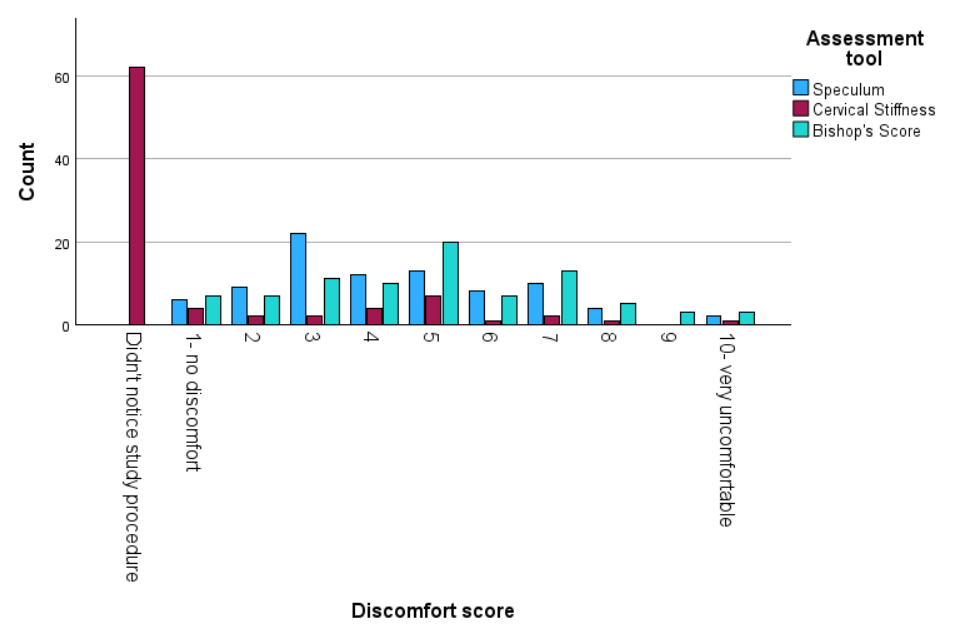

Supplement: Supplementary file 3 — Figure S3: Comparison of Discomfort Score Between Study Procedures. [file BJO-133-1762-s010.docx]
