# Supplementary material for: Objective Cervical Stiffness Assessment Using the Pregnolia System Prior to Induction of Labour: The CASPAR Feasibility Cohort Study
Source: BJOG. 2026 Mar 25;133(9):1762–70. doi: 10.1111/1471-0528.70229 (PMC13419266; doi:10.1111/1471-0528.70229)
Supplement: Supplementary file 4 — Figure S4: Comparison ROC curves between Cervical Assessment Tools for Induction of Labour Binary Clinical Outcomes. [file BJO-133-1762-s003.docx]

**Figure S4**


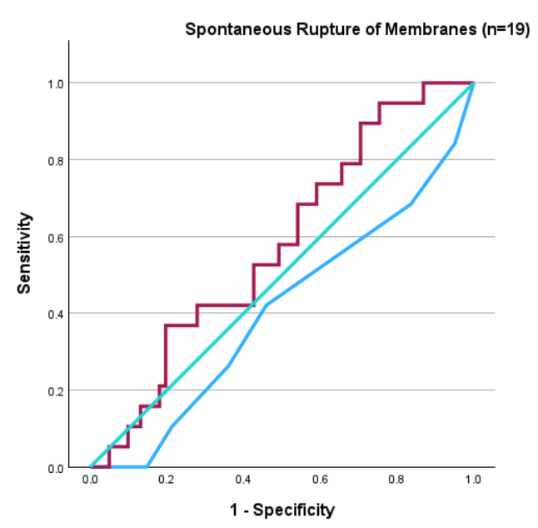

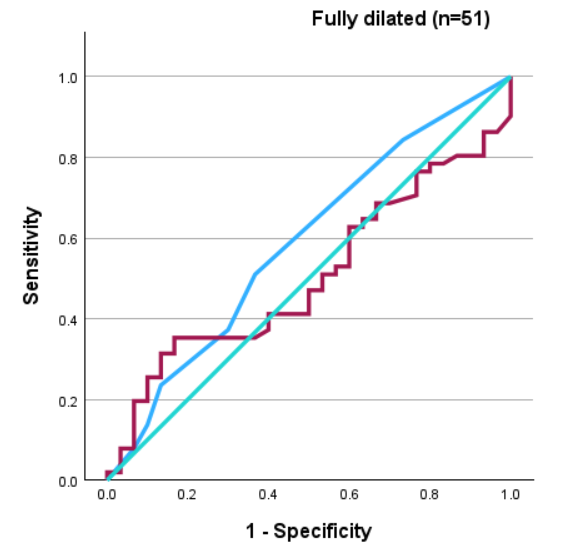

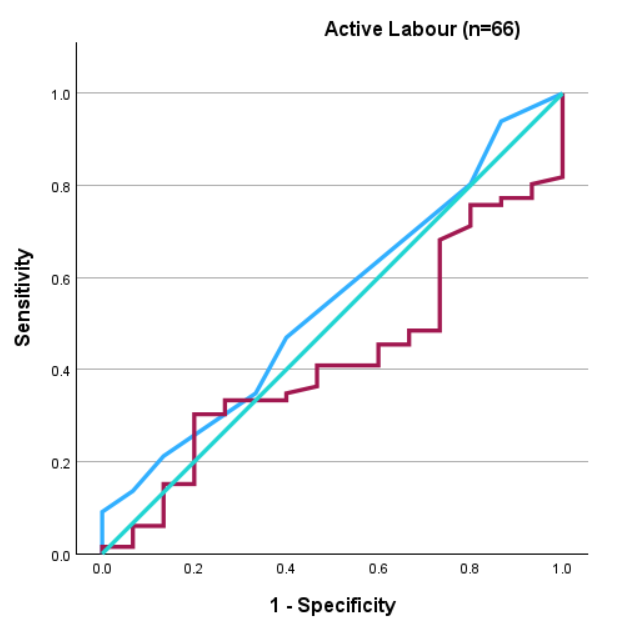
*Comparison ROC curves between Cervical Assessment Tools for Induction of Labour Binary Clinical Outcomes*


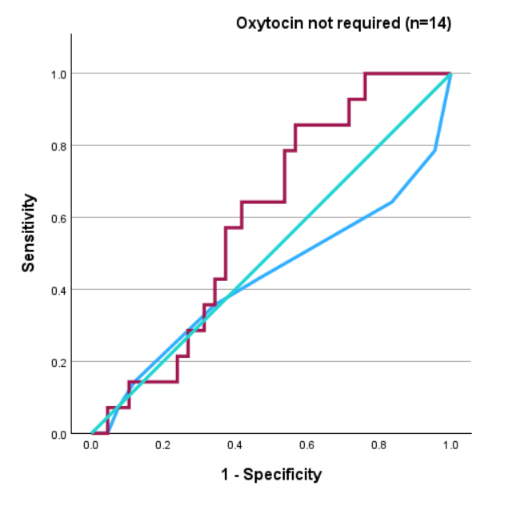
**
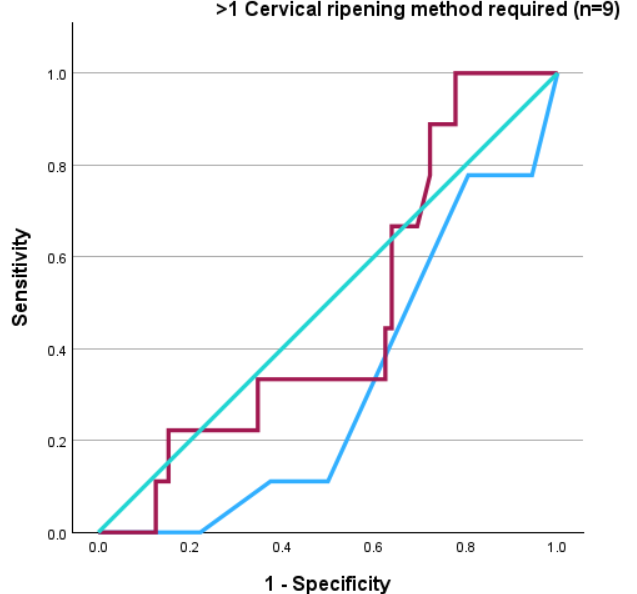
**
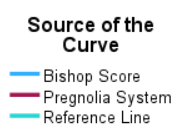


*Note.* Figures involving small numbers (<10) for exploratory analysis and hypothesis generation only. Not intended to demonstrate predictive performance. See Table S5 for associated AUC with 95% CI.
