# Supplementary material for: Objective Cervical Stiffness Assessment Using the Pregnolia System Prior to Induction of Labour: The CASPAR Feasibility Cohort Study
Source: BJOG. 2026 Mar 25;133(9):1762–70. doi: 10.1111/1471-0528.70229 (PMC13419266; doi:10.1111/1471-0528.70229)
Supplement: Supplementary file 5 — Figure S5: Associations between Cervical Assessment Tools and Continuous Outcome of Interest. [file BJO-133-1762-s011.docx]

**Figure S5**

*Associations between Cervical Assessment Tools and Continuous Outcome of Interest*

1. **
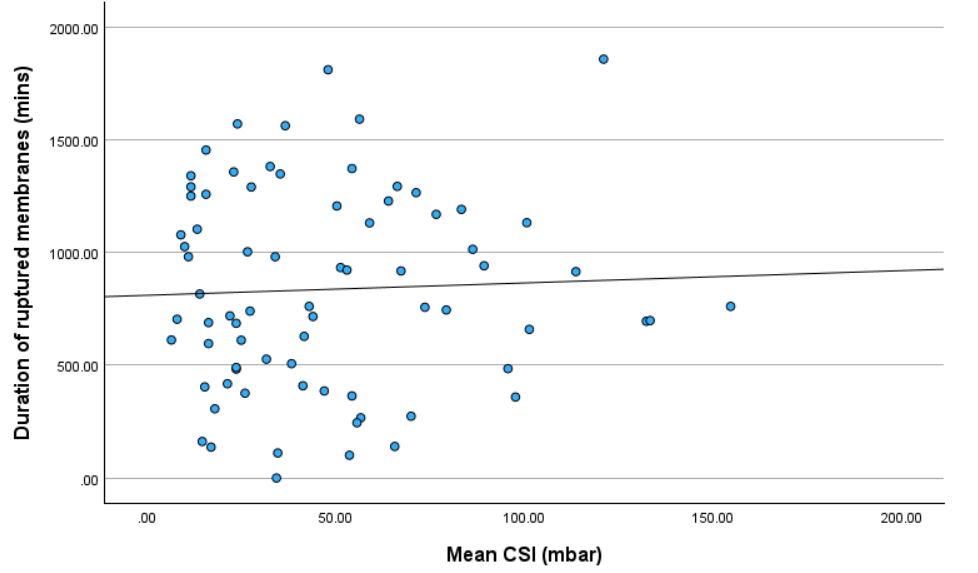
Duration of rupture of membranes**


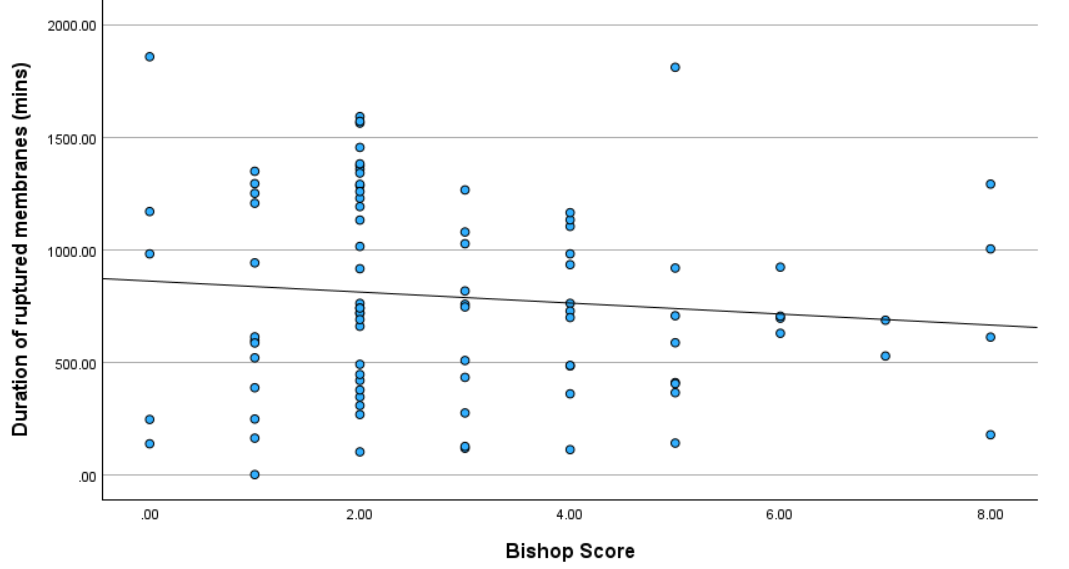


R=0.042 (-0.187, 0.267)

R=-0.109 (-0.309, 0.101)

1. **
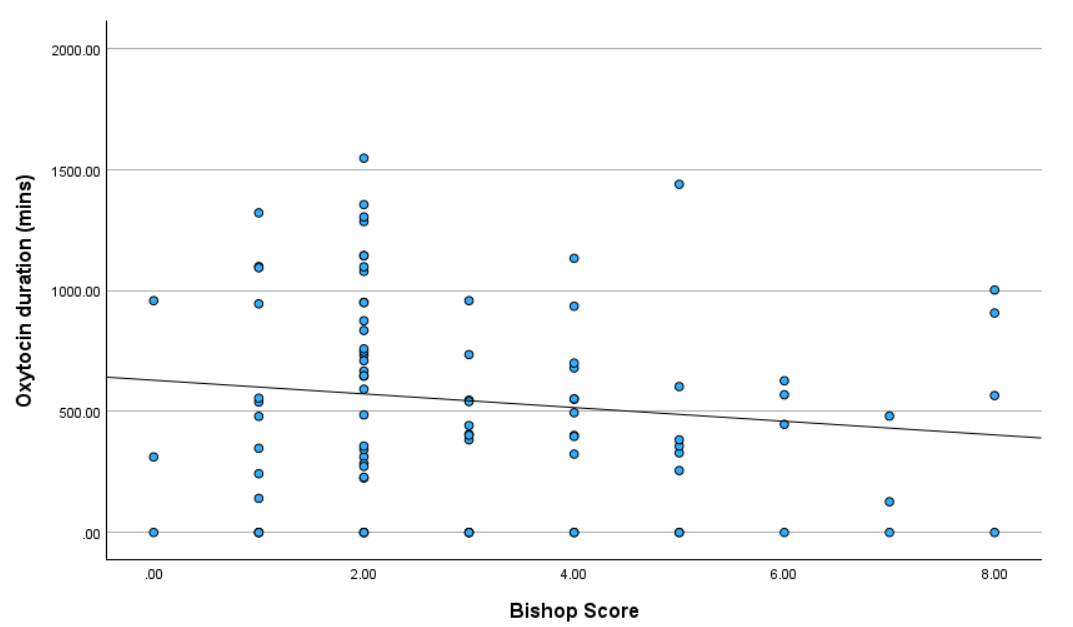

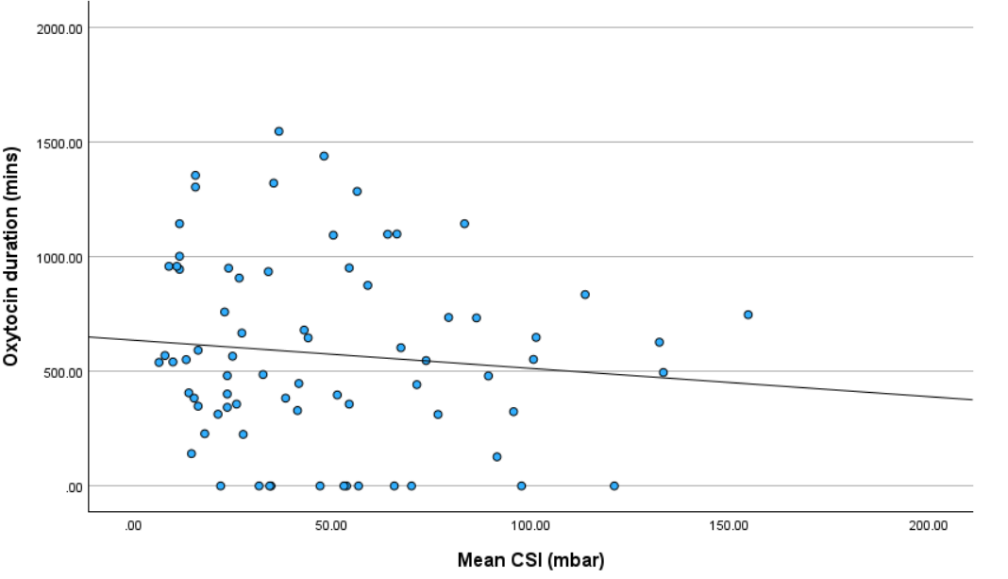
Duration of Oxytocin**

R=-0.136, (-0.334, 0.075)

R=-0.104 (-0.325, 0.127)

1. **Interval from onset of induction until delivery**


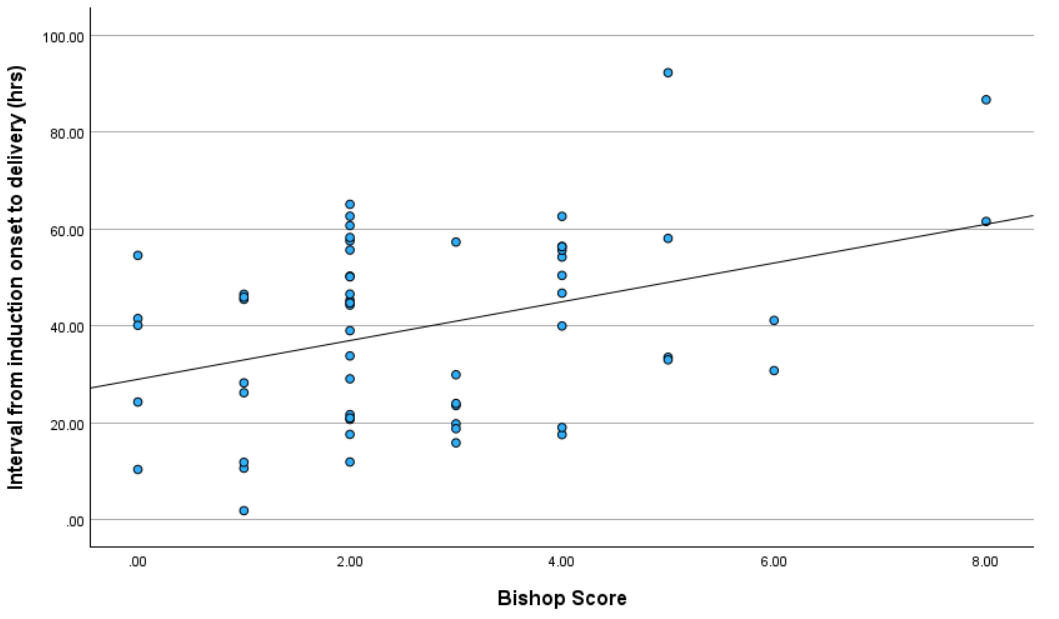

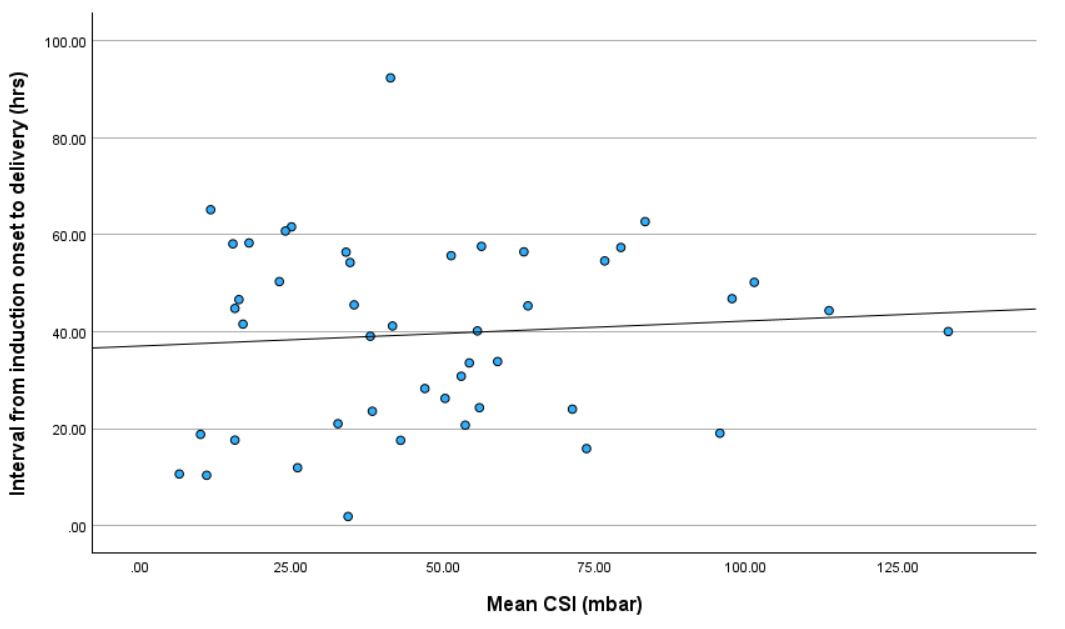


R= 0.376 (-0.131, 0.578)

R= 0.081 (-0.214, 0.363)

1. **
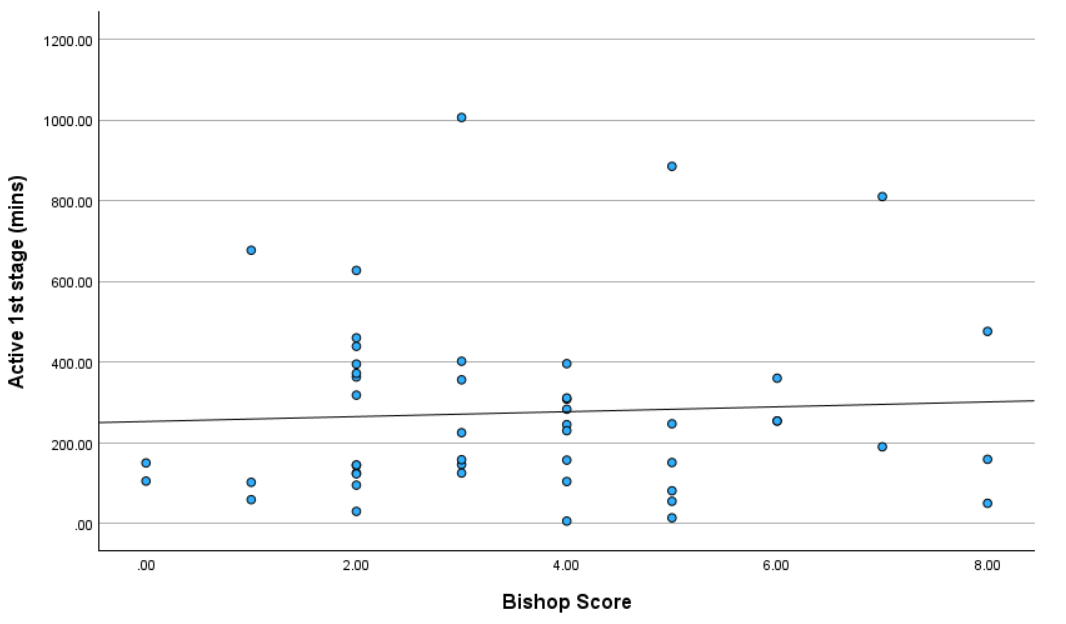
**
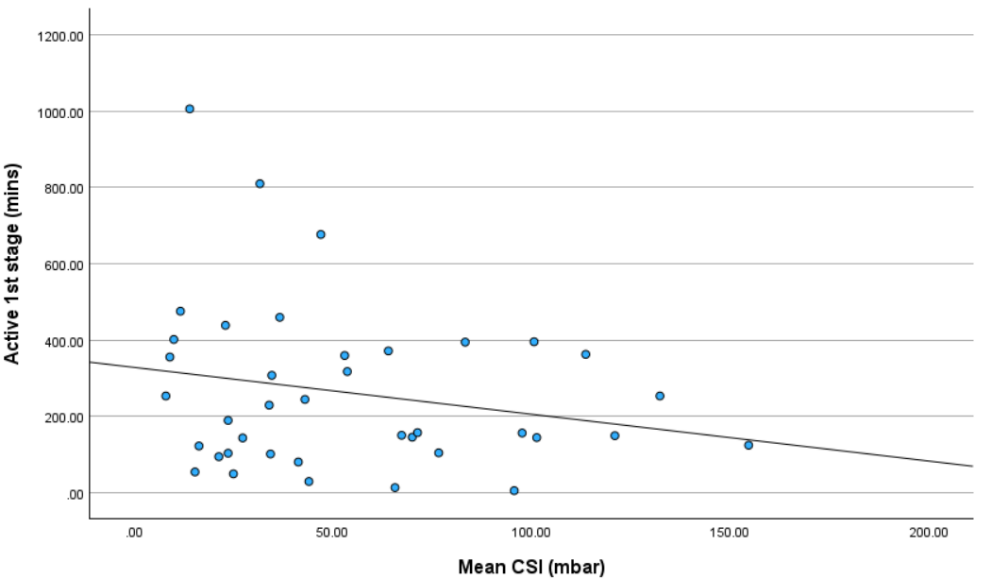
**Duration of active 1^st^ Stage**

R= 0.055 (-0.233, 0.334) 0.107)

R= -0.215 (-0.497, 0.107)
