# Supplementary material for: Objective Cervical Stiffness Assessment Using the Pregnolia System Prior to Induction of Labour: The CASPAR Feasibility Cohort Study
Source: BJOG. 2026 Mar 25;133(9):1762–70. doi: 10.1111/1471-0528.70229 (PMC13419266; doi:10.1111/1471-0528.70229)
Supplement: Supplementary file 6 — Figure S6: Association between Cervical Assessment Tool Results; Bishop Score and Pregnolia System Cervical Stiffness. [file BJO-133-1762-s009.docx]

**Figure S6**

*Association between Cervical Assessment Tool Results; Bishop Score and Pregnolia System Cervical Stiffness*

**
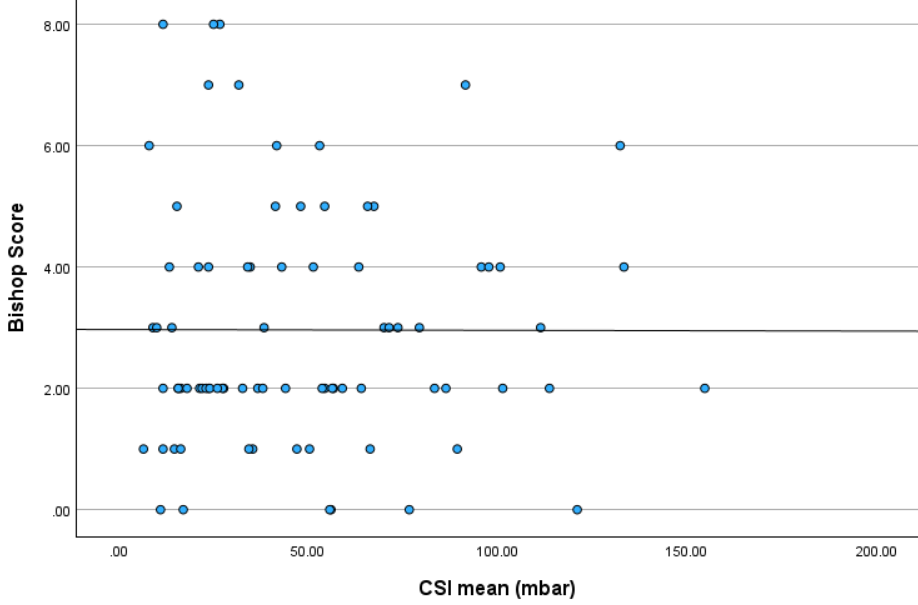
**

R= -0.002 (-0.220, 0.216)
