# Supplementary material for: Objective Cervical Stiffness Assessment Using the Pregnolia System Prior to Induction of Labour: The CASPAR Feasibility Cohort Study
Source: BJOG. 2026 Mar 25;133(9):1762–70. doi: 10.1111/1471-0528.70229 (PMC13419266; doi:10.1111/1471-0528.70229)
Supplement: Supplementary file 7 — Table S1: Participant Questionnaire Study Assessment Discomfort Scores. [file BJO-133-1762-s006.docx]

**Table S1**

*Participant Questionnaire Study Assessment Discomfort Scores*

| **Assessment tool** | **Mean discomfort score** | **Mean difference** | **95% CI** |
| --- | --- | --- | --- |
| Speculum | 4.35 | 3.15 | 2.58, 3.73 |
| Cervical stiffness | 1.20 |  |  |
| Bishop score | 4.93 | 3.73 | 3.09, 4.37 |
| Cervical stiffness | 1.20 |  |  |
| Speculum | 4.35 | 0.58 | 0.05, 1.11 |
| Bishop score | 4.93 |  |  |
