# Supplementary material for: Objective Cervical Stiffness Assessment Using the Pregnolia System Prior to Induction of Labour: The CASPAR Feasibility Cohort Study
Source: BJOG. 2026 Mar 25;133(9):1762–70. doi: 10.1111/1471-0528.70229 (PMC13419266; doi:10.1111/1471-0528.70229)
Supplement: Supplementary file 8 — Table S2: Summary of Reasons for not Achieving Triplicate Measurements. [file BJO-133-1762-s005.docx]

**Table S2**

*Summary of Reasons for not Achieving Triplicate Measurements*

| Reason triplicate measurement not obtained | Number (%) of eligible cohort for cervical stiffness assessment  N=98 |
| --- | --- |
| **Pregnolia probe not placed at speculum**   - Exclusion criteria (bleeding/dilated) - Participant unable to tolerate speculum examination for duration to achieve triplicate measurements | **5 (5%)**  3 (3%)  2 (2%) |
| **Pregnolia probe placed at speculum**   - Exclusion criteria (bleeding) - Unable to achieve adequate seal with Pregnolia probe   - Participant unable to tolerate speculum examination for duration to achieve triplicate measurements | **17 (17%)**  1 (1%)  14 (14%)  2 (2%) |
| **Amended for analysis**   - Measurement discounted at operator discretion (seal concerns) - “low” measurements managed as per manufacturer instruction | **6 (6%)**  4(4%)  2 (2%) |
