# Supplementary material for: Objective Cervical Stiffness Assessment Using the Pregnolia System Prior to Induction of Labour: The CASPAR Feasibility Cohort Study
Source: BJOG. 2026 Mar 25;133(9):1762–70. doi: 10.1111/1471-0528.70229 (PMC13419266; doi:10.1111/1471-0528.70229)
Supplement: Supplementary file 9 — Table S3: Definitions used for Clinical Outcomes. [file BJO-133-1762-s001.docx]

**Table S3**

*Definitions used for Clinical Outcomes*

| ***Clinical Outcome*** | ***Definition used*** |
| --- | --- |
| *Failed Induction of Labour* | *Unable to artificially rupture membranes/never established in labour (≥4cm)- decision by clinical team* |
| *Active Labour* | *Documented as ≥ 4cm within labour documentation.* |
| *Fully dilated* | *Cervical dilatation = 10cm* |
| *Active 1^st^ Stage of Labour* | *Period between ≥4cm and fully dilated (10cm) in labour documentation.* |
| *2^nd^ Stage of Labour* | *Period between fully dilated and delivery of baby.* |
