# Supplementary material for: Objective Cervical Stiffness Assessment Using the Pregnolia System Prior to Induction of Labour: The CASPAR Feasibility Cohort Study
Source: BJOG. 2026 Mar 25;133(9):1762–70. doi: 10.1111/1471-0528.70229 (PMC13419266; doi:10.1111/1471-0528.70229)
Supplement: Supplementary file 10 — Table S4: Feasibility of Collecting Core Outcome Set for Trials on Induction of Labour using CASPAR Protocol. [file BJO-133-1762-s008.docx]

**Table S4**

| **Short-term maternal outcomes** | | **Feasible?** | **Short-term neonatal outcomes** | | **Feasible?** |
| --- | --- | --- | --- | --- | --- |
| Cardio-respiratory arrest | 0 | Yes** | Admission to neonatal unit | 7 (7%) | Yes |
| Damage to internal organs (bowel, bladder or ureters) | 0 | Yes** | Birth trauma | 0 | Yes** |
| Haemorrhage*  Average EBL (mls)  PPH | 625±456  24 (25%) | Yes | Death of the baby | 0 | Yes |
| Hysterectomy for any complications resulting from birth | 0 | Yes** | Hypoxic ischaemic encephalopathy or need for therapeutic hypothermia | 1 (1%) | Yes |
| Intensive care admission | 1 (1%) | Yes | Meconium aspiration syndrome | 0 | Yes** |
| Length of hospital stay (days) | 3±2 | Yes | Need for respiratory support | 3 (3%) | Yes |
| Maternal death | 0 | Yes** | Neonatal infection* | 0 | Yes** |
| Maternal infection *  Commenced on sepsis pathway  Positive blood cultures | 10 (10%)  0 | Yes* | Neonatal seizures | 1 (1%) | Yes |
| Maternal satisfaction | N/A | No |  | | |
| Mode of delivery  Vaginal  caesarean | 57 (59%)  40 (41%) | Yes |  |  |  |
| More than one induction agent required | 12 (12%) | Yes |  |  |  |
| Oxytocin augmentation | 72 (74%) | Yes |  |  |  |
| Postnatal depression | N/A | No |  |  |  |
| Pulmonary embolus | 0 | Yes** |  |  |  |
| Stroke | 1 (1%) | Yes |  |  |  |
| Time from induction of labour to delivery (hrs) | 41±18 | Yes |  |  |  |
| Uterine hyperstimulation*  Terbutaline use  Interruption of oxytocin | 27 (28%)  6 (6%)  21 (21%) | Yes* |  |  |  |
| Uterine scar dehiscence or rupture | n/a- all primps | No |  |  |  |
| **Long term maternal outcomes** | |  | **Long term neonatal outcomes** | |  |
| Need for operative pelvic floor repair | N/A | No | Long term disability including neurodevelopmental delay | N/A | No |

*Feasibility of Collecting Core Outcome Set for Trials on Induction of Labour using CASPAR Protocol*

*Note.* Continuous variables represented as Mean ±SD as normally distributed and categorical variables represented as number (%).

***No definitions provided by Delphi consensus. Following definitions used: Haemorrhage; >500mls vaginal delivery, >1000mls caesarean, Maternal infection; commenced on sepsis pathway and/or positive blood cultures, Uterine hyperstimulation; use of terbutaline, or interruption of oxytocin, Neonatal infection; positive blood cultures or documented rise in CRP.

** No cases in small dataset, therefore not necessarily robustly tested ability to capture relevant data.
