# Supplementary material for: Objective Cervical Stiffness Assessment Using the Pregnolia System Prior to Induction of Labour: The CASPAR Feasibility Cohort Study
Source: BJOG. 2026 Mar 25;133(9):1762–70. doi: 10.1111/1471-0528.70229 (PMC13419266; doi:10.1111/1471-0528.70229)
Supplement: Supplementary file 11 — Table S5: Diagnostic Performance for Cervical Assessment Tools for each Binary Outcome of Interest. [file BJO-133-1762-s004.docx]

**Table S5**

*Diagnostic Performance for Cervical Assessment Tools for each Binary Outcome of Interest*

| **Clinical Outcome** | **N** | **PS AUC** | **95% CI** | **BS AUC** | **95% CI** |
| --- | --- | --- | --- | --- | --- |
| Vaginal delivery | 47 | 0.466 | 0.340, 0.593 | 0.621 | 0.497, 0.745 |
| Failed IOL | 7 | 0.563 | 0.347, 0.780 | 0.463 | 0.239, 0.687 |
| Active labour | 66 | 0.544 | 0.387, 0.702 | 0.423 | 0.277, 0.570 |
| Fully dilated | 51 | 0.505 | 0.378, 0.631 | 0.586 | 0.457, 0.716 |
| Spontaneous Rupture of Membranes | 19 | 0.577 | 0.439, 0.715 | 0.406 | 0.259,0.552 |
| No oxytocin required | 14 | 0.600 | 0.464, 0.737 | 0.434 | 0.246, 0.622 |
| >1 cervical ripening method required | 9 | 0.474 | 0.298, 0.649 | 0.316 | 0.163, 0.469 |

*Note*. PS: Pregnolia System, BS: Bishop Score, AUC: Area under the curve.

AUC <.5 demonstrates poor performance, meaning no better than chance.

See Figure 4 for associated ROC for each clinical outcome.
